# Study of the Influence of Winding and Sensor Design on Ultra-High Frequency Partial Discharge Signals in Power Transformers

**DOI:** 10.3390/s20185113

**Published:** 2020-09-08

**Authors:** Chandra Prakash Beura, Michael Beltle, Stefan Tenbohlen

**Affiliations:** Institute of Power Transmission and High Voltage Technology (IEH), University of Stuttgart, 70569 Stuttgart, Germany; michael.beltle@ieh.uni-stuttgart.de (M.B.); stefan.tenbohlen@ieh.uni-stuttgart.de (S.T.)

**Keywords:** power transformers, partial discharge, PD, UHF, monitoring, PD sensors, simulation, sensitivity evaluation

## Abstract

Ultra-high frequency (UHF) partial discharge (PD) measurements in power transformers are becoming popular because of the advantages of the method. Therefore, it is necessary to improve the basic understanding of the propagation of signals inside the transformer tank and the factors that influence the sensitivity of the measurement. Since the winding represents a major obstacle to the propagation of the UHF signals, it is necessary to study the effect of winding design on signal propagation. Previous research activities have studied these effects using simplified models, and it is essential to consider the complexity of propagation in a complete transformer tank. Additionally, the quality of UHF PD measurements depends, to a large extent, on the sensitivity of the UHF sensors. In this contribution, a simulation model consisting of a simple, grounded enclosure with multiple winding designs is used to study the propagation characteristics of UHF signals when an artificial PD source is placed inside the winding. After analysis of the results, the winding designs are incorporated in an existing and validated simulation model of a 420 kV power transformer and analyzed to observe the influence in a more complex structure. Two commonly used sensor designs are also used in the simulation model to receive the signals. In all cases, the propagation and signal characteristics are analyzed and compared to determine the influence of the winding and sensor design on the UHF signals. It is found that the level of detail of winding design has a significant impact on the propagation characteristics. However, the attenuation characteristics of the UHF signals received by the two sensor designs are similar, with the electric field distribution around the sensor being the key difference.

## 1. Introduction

Ultra-high frequency (UHF) partial discharge measurement in power transformers is performed by using UHF sensors to measure electromagnetic (EM) waves that are generated during the partial discharge (PD) event and then propagate inside the tank until reaching the sensors. The method enjoys certain advantages over traditional electrical PD measurement, such as the possibility of three-dimensional source localization and continuous online monitoring [1,2]. However, improving localization accuracy requires understanding the propagation paths inside transformers, which are quite complex. The active parts of the transformer, such as the core, winding, etc., act as obstacles to the propagation of UHF signals [3]. The PD inside transformer windings, especially, the high-voltage (HV) windings is one of the most commonly occurring faults [4]. Therefore, it is important to investigate the propagation of UHF signals when a PD occurs inside a winding. A winding in power transformers is generally of two types: layer winding and disk winding. The former is used in low-voltage (LV) windings, and the latter is used in medium-voltage (MV) and HV windings [5,6]. It is difficult to perform experimental tests on power transformers with large ratings, where known PD sources are inside the winding, and hence, electromagnetic simulations are used to study electromagnetic propagation [7]. However, the winding design is such simulations is generally simplified to reduce the computational requirements. For example, individual turns of layer windings are not modeled, and the winding is simply modeled as a solid cylinder. For disk windings, the individual turns in a disk are not modeled. From these simplifications, it is apparent that oil-filled gaps between conductors are generally ignored [8,9,10]. Moreover, variations in winding designs are not considered, and the impact of the variation of design on electromagnetic wave propagation is an area that requires investigation. For example, the MV windings may or may not have an air gap between two successive disks, which can influence the propagation of the EM waves.

However, another factor is the design of the UHF sensor itself. Several UHF sensor designs with various strengths have been developed for use in transformers and gas-insulated switchgear (GIS) [11,12,13,14]. In this contribution, two popular sensor designs, which are commercially available, are studied. Based on the installation mechanism, UHF sensors can be classified into two types: drain valve sensors and window-type sensors [15]. The former is directly inserted into the oil volume of the transformer tank using a DIN 50 or DIN 80 drain valve. The latter is installed on a dielectric window, which is available on the tank wall; i.e., the sensor is not immersed in oil. Other differences include the insertion depth of the sensor, which can be varied in the former but is fixed in case of the latter [16]. Positioning UHF sensors in areas where the field stress generated by the 50/60 Hz supply is low is required to prevent unintended PD events [17,18]. Any differences in the characteristics of the signals received and the electric field distribution around the two types of sensors are aspects that require further investigation.

In this contribution, different winding designs are modeled in CST Studio and simulated with artificial PD sources inside to study the influence of winding design on the propagation of the electromagnetic waves. Time-domain electromagnetic simulations are performed on three winding designs, which are first simulated within a smaller metallic enclosure for comparison in a less computationally intensive manner. Then, the simulations are performed with selected winding designs placed inside the tank of a 420 kV transformer. The results obtained are analyzed to determine the influence of the winding design on the obtained signals when PDs are occurring inside the winding.

Additionally, the two sensor types are studied using numerical simulations in CST Microwave Studio. The sensors are used in a validated simulation model of a 300 MVA, 420 kV grid coupling transformer [19], and time-domain electromagnetic field simulations are carried out to observe the characteristics of the signals received by the two designs and analyze the electric field distribution around the sensors.

## 2. Simulation Model

### 2.1. Winding Design

**Figure 1:**
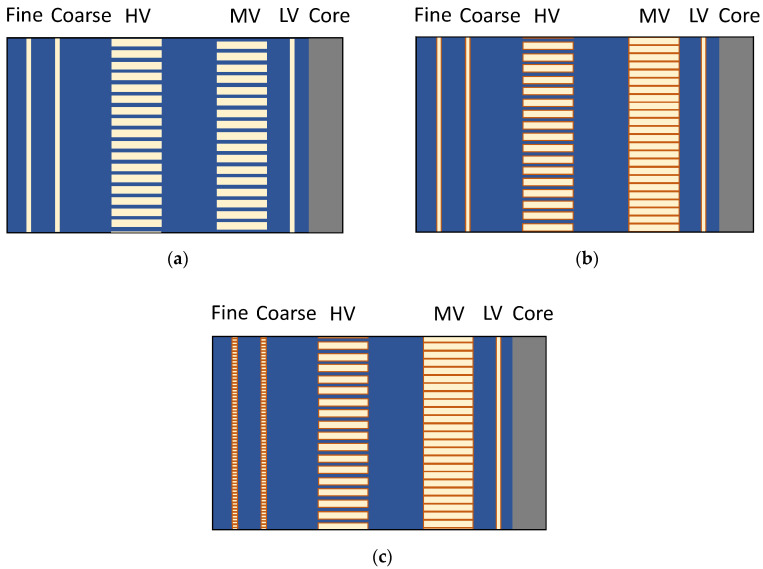
Cross-sectional view of the different winding designs (conductors in yellow, oil in blue, and oil-impregnated paper in orange). (**a**) Reference winding design with insulation layer defined as a coating (not visible); (**b**) Second winding design with reduced oil-filled gaps in the medium-voltage (MV) winding; (**c**) Third winding design with individually modeled turns in the coarse and fine windings.

The evaluated transformer winding design consists of a single layer LV winding, MV disk winding, HV disk winding, single layer coarse winding, and single layer fine winding. There are three different winding designs that have been simulated separately, as shown in Figure 1. However, there are some common factors among all designs: The LV winding is simplified as a solid cylinder. It is closest to the core, and the propagation of the UHF EM waves toward the core is not of interest, since only the waves that propagate out of the winding will be received by the sensors. In addition, the individual turns on one disk of the HV windings are not modeled. The EM waves cannot propagate through these turns, and modeling each turn would be computationally expensive.

The first winding design, as shown in Figure 1a, uses all the usual simplifications, i.e., the layer windings are modeled as solid cylinders. Additionally, the oil-impregnated paper insulation on the winding is specified as a coating of 0.3 mm in the material properties, i.e., the insulation layer is not modeled separately but is automatically considered by CST and is not visible in the model. In this winding model, both the HV and MV winding have an oil-filled gap between two adjacent disks. The first winding model will be used as a reference to compare the second and third models, as it is simulating the worst-case scenario in which the EM waves cannot propagate through the outer layer windings.

The second winding model, as shown in Figure 1b, removes the oil-filled gap between the adjacent disks of the MV winding. Additionally, in this model, the oil-impregnated paper insulation over the conductors is explicitly modeled and not a part of the material properties. As expected, this model is more computationally expensive than the first two models because of the mesh required for the thin layers of paper.

The third winding model is the same as the second, but the coarse and fine windings are not simplified as solid cylinders. Each turn of the layer winding along with its associated oil-impregnated paper insulation is explicitly modeled, as shown in Figure 1c. This model is the most computationally expensive of all models, resulting in more than three times the number of cells compared to the reference winding design.

### 2.2. Sensor Design

**Figure 2:**
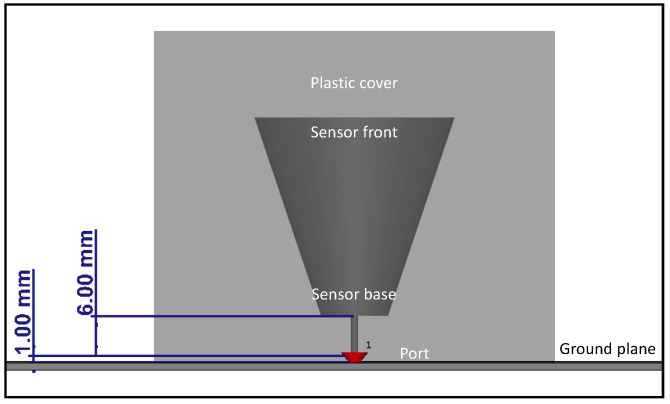
Design of the drain valve sensor.

The drain valve sensor consists of a sensor head in the shape of a conical frustum with a conductor connected to the base of the sensor head, and the sensor head is covered with a plastic cover with a relative permittivity of *ε_r_* = 4. The conductor is used for port definition, as will be explained in this chapter. The design of the sensor is shown in Figure 2. The same conical frustum design is retained for the window-type sensor.

**Figure 3:**
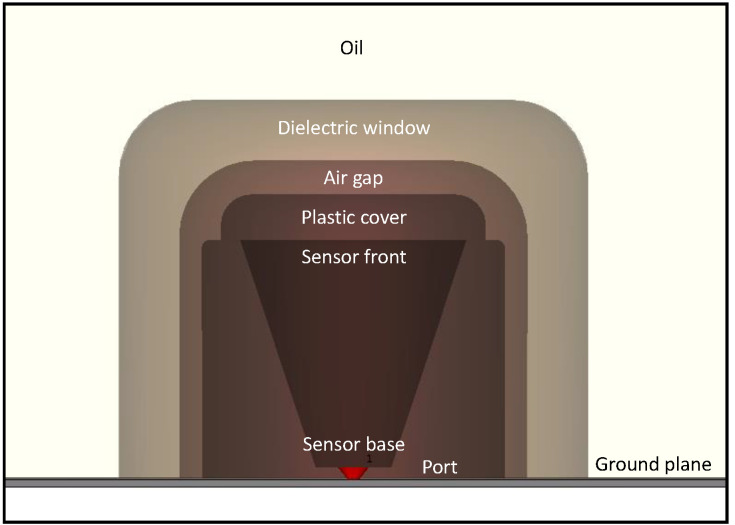
Simulation setup of the window-type sensor in oil.

However, a dielectric window, in which the window-type sensor is placed, is added to the model [20]. The complete sensor with the dielectric window is shown in Figure 3. Additionally, the material of the window is given the same relative permittivity as that of the oil (*ε_r_* = 2.3), since the actual value is close to that of mineral oil. All metal objects are modeled as Perfect Electric Conductors (PECs). The dimensions of the plastic cover on the sensor head were also adapted based on measured values to fit the sensor inside the dielectric window. However, the sensor is not flush with the inner surface of the dielectric window: there is an air gap between the outer surface of the plastic cover on the sensor head and the inner surface of the dielectric window.

For the drain valve sensor, the base of the 6 mm long conductor is placed 1 mm above the transformer tank wall, which acts as the ground plane, as shown in Figure 2, and the same is done for the window-type sensor. A discrete port is defined between the base of the conductor and the ground plane and is used to provide the excitation signal to the setup, as shown in Figure 2 and Figure 3 [21].

### 2.3. Test Setups

**Figure 4:**
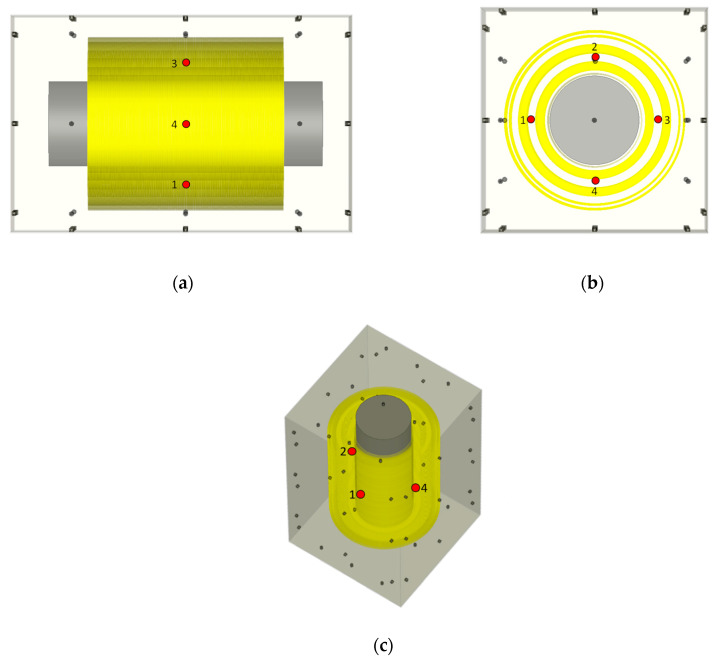
Simplified single-phase model with nine sensors on each face (gray circles) and four partial discharge (PD) sources (red circles) inside the winding (**a**) Side view; (**b**) Top view; (**c**) Isometric view.

There are two types of test setups used for the transient simulations: a reduced setup to investigate the influence of the windings and a full transformer setup. The first is a simplified single-phase model of the transformer that consists of only one phase winding block, as shown in Figure 4, which was created to study the signal propagation characteristics and reduce the computational requirements for simulation. The width and height of this cuboidal metallic enclosure correspond to that of the 420 kV transformer. Since only a one-phase winding block is placed in the enclosure, the length of the enclosure is approximately one-third of the length of the transformer tank. Four artificial PD sources are placed inside each winding model at the center of the winding height in between the MV and HV winding, as shown in Figure 4. Each face of the enclosure contains 9 sensors in a 3 × 3 grid, resulting in 54 sensors in total. The three winding designs are placed in this simplified single-phase model, and the signal characteristics are studied. Figure 4 shows the simplified-single phase model with the third winding design (Figure 1c).

**Figure 5:**
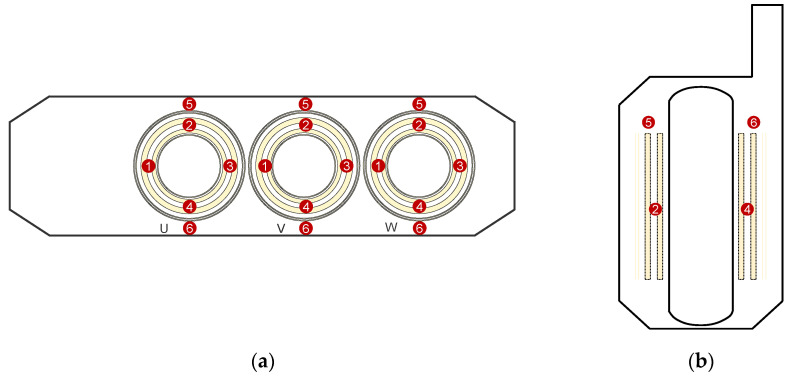
Location of the 18 artificial PD sources inside the transformer model. (**a**) Top view; (**b**) Side view.

The second setup represents a complete transformer model which consists of 18 possible PD source locations with 12 inside and six outside the winding, as shown in Figure 5. The six sources outside the windings correspond to the locations of the lead exits. Considering a single phase (U, V, or W), the four sources inside the winding are numbered from 1 to 4, and the two sources corresponding to the lead exits are numbered 5 and 6.

**Figure 6:**
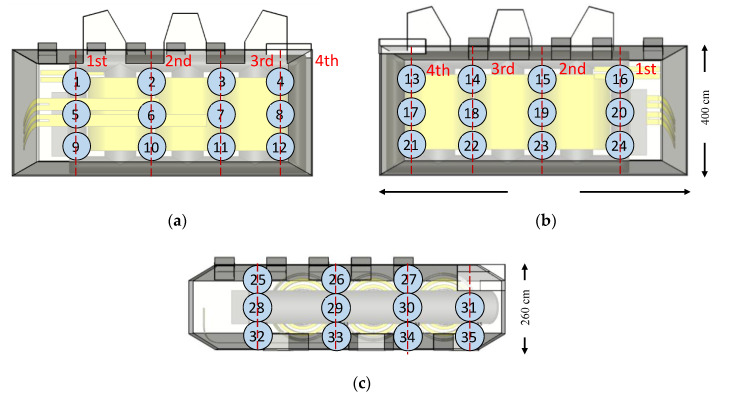
Complete transformer model with 35 ultra-high frequency (UHF) sensors for measurement with blue circles representing sensor positions and red lines representing cutting planes. (**a**) Front view; (**b**) Top view; (**c**) Rear view of transformer tank.

The transformer tank walls consist of 35 sensors for measurement arranged in a grid, as described in [22] and shown in Figure 6. Sensors 1–12 are categorized as the ‘Front’ sensors, Sensors 13–24 are characterized as the ‘Rear’ sensors, and Sensors 25–35 are characterized as the ‘Top’ sensors based on the tank walls on which they are positioned.

For studying the attenuation characteristics inside the transformer, all 18 sources are used. In these simulations, the drain valve sensor model is used in conjunction with the reference winding design. The analysis of the influence of the winding design is done using the simplified single-phase model and the complete transformer model. First, all three winding designs introduced in Figure 1 are analyzed using the four sources in the simplified single-phase model. Then, the third winding model is used in the complete transformer model with PD sources at two positions U1 and W3 (because of computational cost) and compared with the corresponding results obtained from the reference winding in the transformer.

Positions U1 and W3 are selected because they are inside the winding, resulting in indirect propagation to receiving sensors. Additionally, they are located on the opposite ends of the tank, thus providing the longest propagation paths to the receiving sensors farthest from them when compared to the other sources. Information about the longest propagation paths is necessary to evaluate the distance-dependent attenuation in the transformer.

For analyzing the influence of the sensor design, the complete transformer model with the reference winding design is used, and the 12 sources inside the winding are simulated. The sources outside the windings are not considered because of their comparatively simpler propagation paths. The simulation for each PD source is run twice: once with the drain valve sensors for measurement and next with the window-type sensors. All transient simulations are performed with oil (having complex permittivity) as the dielectric medium in the tank [22]. The simulation characteristics remain unchanged from the referenced work, and only the winding designs are changed. Therefore, these aspects of the simulation are not reiterated in the present work.

## 3. Results

### 3.1. Influence of Winding Design

#### 3.1.1. Simplified Single-Phase Model

The results obtained from the simulations provide information about the electromagnetic wave propagation in the three-dimensional space. The key aspect to be observed in the results is whether the EM waves propagating through the oil-impregnated paper layers [3,23] can significantly impact the time of arrival (ToA) and attenuation of the signals at the different sensors.

**Figure 7:**
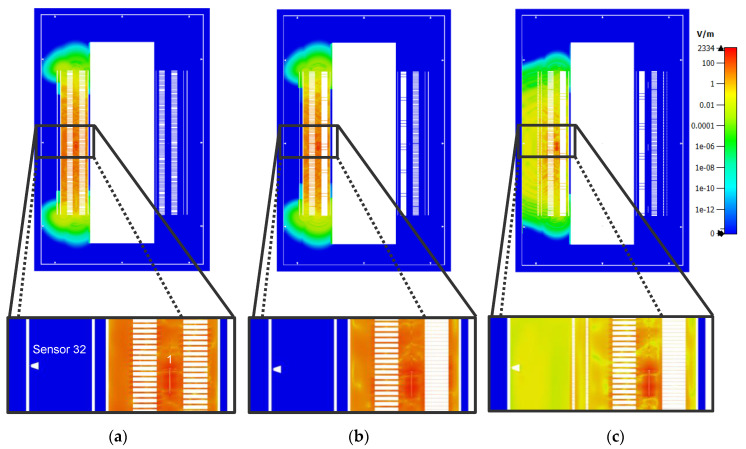
Electromagnetic wave propagation in the simplified single-phase model for the different winding designs at time *t* = 7 ns after triggering of PD source at position 1. (**a**) Reference winding design; (**b**) Second winding design; (**c**) Third winding design.

The propagation of the signals in the reference winding model is as follows. In the case of the PD source at position 1 (as shown in Figure 4), the signals propagate toward the outer and inner layer windings where they are reflected toward the top and bottom of the winding block. Therefore, the sensors installed on the top and bottom of the enclosure have the highest signal strengths and the fastest ToAs. As shown in Figure 7a, the EM waves exit the winding at the top and the bottom at around 7 ns after the PD source is triggered. The signals reach sensor 32, which is at the center of the tank face, after approximately 16 ns.

In the second winding model, it is observed that the propagation characteristics remain essentially unchanged with respect to the reference winding model, as shown in Figure 7b. The significantly reduced gaps between the consecutive disks of the MV winding does not seem to impact the propagation of the EM waves toward the inner layer winding. Hence, the electric field distribution inside the winding block looks similar to that of the reference model. The time taken for the EM waves to exit the winding from the top and the bottom remains unchanged along with the calculated ToAs at each UHF sensor.

Significant differences can be observed on comparing the reference model to the third model. The electric field strength inside the winding is lower in the latter at the same time instant, as shown in Figure 7c, since the EM waves can now propagate through the oil-impregnated paper layer between each turn of the outer layer windings. For the same reason, the time taken for the EM waves to exit the winding from the top and the bottom is increased. It is important to stress that the waves do not travel slower because they pass outwards through the outer layer windings. However, it takes more time for the signal at the top and bottom to reach the same magnitude as they had before because the initial wavefront can disperse more freely and is less intense by the time it reaches those positions. As can be observed in Figure 7c, at 7 ns after the triggering of the PD source, the EM waves are just reaching the ends of the winding height. However, at the same time, the EM waves have propagated radially outwards and reached sensor 32. Compared to the ToA of 16 ns in the reference model, a ToA of approximately 6–7 ns represents a reduction of approximately 64%. This reduction is not observed for all sensor positions, but on average, the ToAs are 10% quicker in the third model.

The comparison of the different winding models shows that the reference and second designs have similar ToAs, whereas the third design has quicker ToAs. From the observations provided by the simplified single-phase model, the extra computational expenses incurred by the third winding model are necessary because the influence of the gaps cannot be neglected for broadband UHF signal propagation; i.e., the first two winding designs are too simple. However, a simplified single-phase model does not represent the complexity of an actual transformer.

Therefore, in the next step, the reference winding and the third winding model are placed in the simulation model of a 420 kV transformer, and the signal characteristics are studied to determine if the differences still occur inside a much larger enclosure with much more complex propagation paths. However, the complexity of the third design results in approximately 200 million mesh cells compared to the 62 million that results from the reference design. As a result of the sheer computational time required, all sources could not be tested with the third winding design. The PD sources at positions U1 and W3 (both inside the respective windings) are considered when evaluating the third winding design.

#### 3.1.2. Power Transformer Model

Before analyzing the distance-dependent attenuation, it is necessary to distinguish between Line-of-Sight (LoS) distance and propagation distance. The LoS distance is simply a straight line drawn from the source to the receiver and ignores any obstacles that prevent the propagation of the UHF signals, whereas the propagation distance is the actual path traveled by the UHF signals. The propagation distance is calculated from the Time of Arrival (ToA) of the UHF signals at the receiving sensors and the speed of signal propagation in oil (assumed as two-thirds the speed of light). The ToA was determined by using the energy criterion [24]. The energy criterion takes the energy (*S_i_*) of the UHF signal at a time instant (*i*) and adds a negative trend (*δ*) to the value, thus providing a quantity called the partial energy ($S_{i}^{’}$). The time instant at which the partial energy plot reaches its global minima provides the ToA of the UHF signals. The partial energy is calculated using Equation (1),

(1) $$S_{i}^{’}=S_{i}-i\delta =\overset{i}{\underset{k=0}{\sum }}\left(u_{k}^{2}-i\delta \right)$$

where the negative trend (*δ*) is calculated from Equation (2) and uses the values of the total energy of the signal (*S_N_*), length of the signal (*N*), and a parameter *α* = 1.

(2) $$\delta =\frac{S_{N}}{\alpha \cdot N}$$

**Figure 8:**
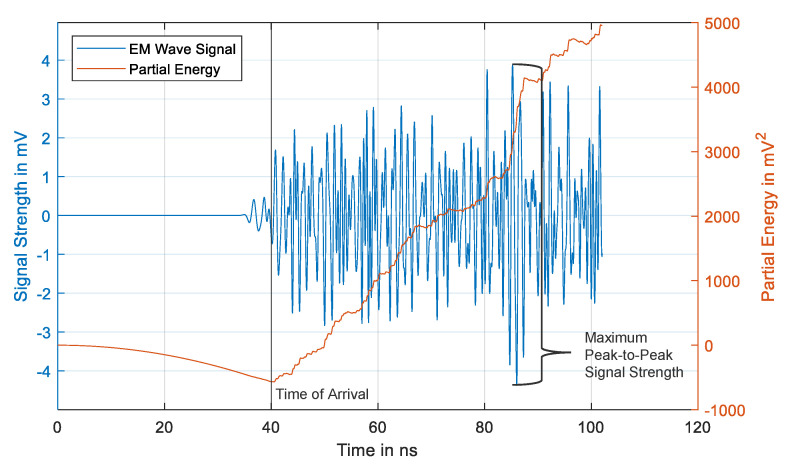
The time-domain signal received by a UHF sensor during a simulation run with the partial energy curve.

In Figure 8, a time-domain waveform of the UHF signal received by a UHF sensor during a simulation run is shown. The partial energy curve is also displayed along with a line representing the ToA determined by the energy criterion. Additionally, signal strength refers to the maximum peak-to-peak voltage of the time-domain waveform.

**Figure 9:**
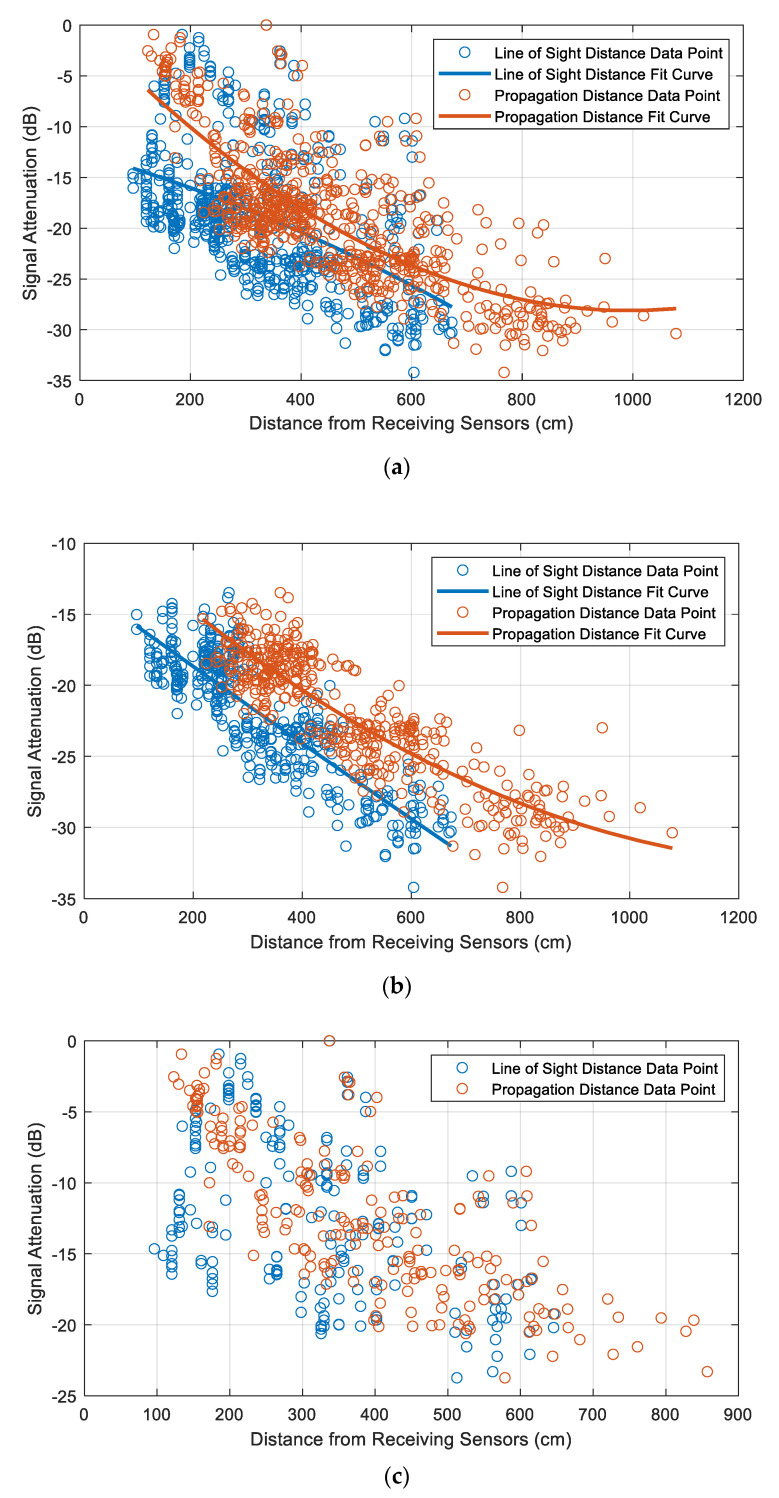
Comparison of Line-of-Sight distance and propagation distance-dependent attenuation in the transformer model using the reference winding design (**a**) All artificial PD sources; (**b**) PD sources inside winding; (**c**) PD sources outside winding.

Since the LoS distance-dependent attenuation is a simplification, it is necessary to evaluate whether it provides an accurate representation of the attenuation inside the tank. All 18 sources (12 inside the winding and 6 outside the winding) were simulated in the model of the transformer using the drain valve sensor model and the reference winding design. The attenuation curve was plotted as shown in Figure 9a. A quadratic function was used to fit the data points. It was observed that the LoS fit curve was a much poorer fit than the propagation distance fit curve on evaluating the sum of squares due to error (SSE), which shows how useful the fit is for prediction. However, for both fit curves, there are a lot of data points exhibiting significant deviation from the fit. Therefore, the data points were split into two categories based on the location of the PD sources, namely ‘Inside Winding’ and ‘Outside Winding’. The former category consists of the data from the 12 PD sources inside the winding, and the latter consists of the data from the 6 PD sources outside the winding from Figure 5. The fit curves for these two categories are shown in Figure 9b,c. It can be observed that the fit significantly improves for the ‘Inside Winding’ category, as shown in Figure 9b. A clear demarcation can be observed between the data points representing each type of distance measurement. However, the fit remained poor for the ‘Outside Winding’ category, as shown in Figure 9c. In addition, for the ‘Outside Winding’ category, the demarcation between the data points is not as distinct as in the case of the ‘Inside Winding’ category. There is more overlap in the values, suggesting that in the case of PD sources outside the winding, the LoS distance and the propagation distance are approximately equal. Another observation is that for similar propagation distances, the attenuation is higher when the source is inside the winding. It can also be observed from Figure 9b that the attenuation curve for the propagation distance is effectively the LoS attenuation curve shifted along the *x*-axis by approximately 120 cm, which corresponds to half of the winding height, i.e., the distance that the UHF signal travels to exit the winding. In Figure 9a–c, 0 dB corresponds to the highest measured signal strength.

**Figure 10:**
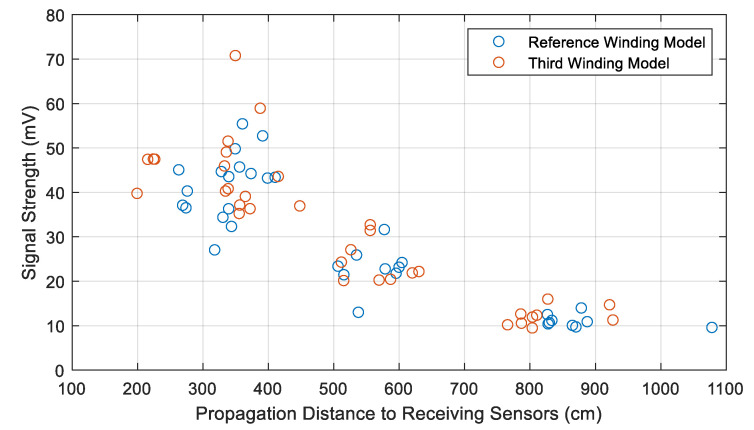
Comparison of signal attenuation between the reference and third winding designs at different propagation distances with the source at position W3.

In the next step, the simulation was run using the third winding design with the sources at positions U1 and W3. From the attenuation curves for both sources (U1 and W3) and on comparing with the reference design, it was observed that the signal strengths are approximately equivalent at comparable propagation distances for both designs. Only a few receiving sensors record comparatively higher signal strengths in the third winding design, as shown in Figure 10 for source W3. On average, obtained signal strengths were found to be approximately 8% higher in the case of the third winding design. Statistically, for the third winding model, the propagation distance is shorter on average, which means that the ToAs are quicker compared to the reference model.

These findings correspond closely to those observed from the simplified single-phase model. The ‘Top’ sensors, on average, receive 20% higher signal strengths (on comparing the peak-to-peak signal strengths in mV) in the third winding design compared to the reference design, which is the biggest improvement. The ‘Front’ and ‘Rear’ receivers show a 2–3% increase in peak-to-peak signal strengths. The explanation for the increased signal strengths received by the ‘Top’ sensors is the reduced gap between the adjacent disks of the MV winding, which results in better directivity toward the ‘Top’ sensors. For example, considering the source at position W3 and receiver at position 31 (almost directly above the source), in the third winding design, the maximum signal strength occurs almost simultaneously at the ToA. However, in the case of the reference winding design, the maximum signal strength occurs significantly later, when the reflected signals reach the sensor.

**Figure 11:**
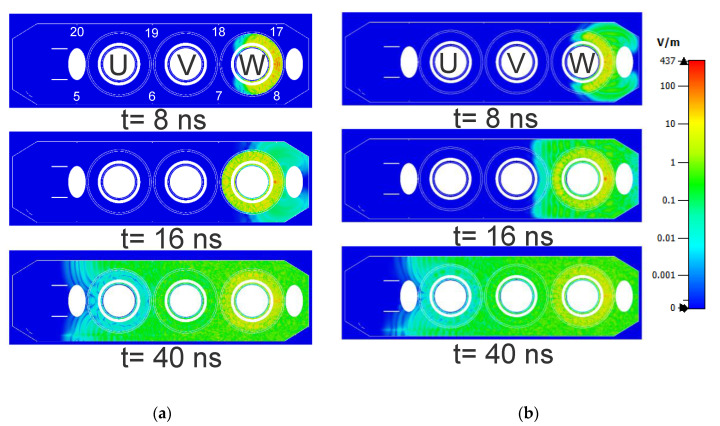
Comparison of electromagnetic wave propagation (shown: absolute electric field strength) inside the transformer at different time instants with the source at position W3. (**a**) Reference winding design; (**b**) Third winding design.

In Figure 11, the electromagnetic wave propagation is shown for the reference and third winding designs at different time instants after the PD source (at position W3) has been triggered. The cutting plane shown in Figure 11 consists of sensors 5, 6, 7, and 8 on the front tank wall and sensors 17, 18, 19, and 20 on the rear tank wall.

- At *t* = 8 ns, in the third winding design, the EM waves have already exited the winding through the gaps in the outer layer windings and reached the closest sensors (sensors 8 and 17). However, in the reference design, the EM waves are still inside the winding and hence, they take a longer time to reach the closest sensors.
- At *t* = 16 ns, in the third design, the EM waves have reached the central winding (winding block V) and propagated through the gaps in the outer layer windings. Whereas in the reference winding design, the EM waves have just exited winding block W and reached the closest sensors to the left of the winding.
- As time elapses and the EM waves propagated along the length of the tank, the differences between the two cases become less pronounced. For example, at *t* = 40 ns, there is no marked difference in the propagation between the two winding designs.

Overall, the biggest influence on ToA is the modeling of the outer layer windings, which allow the signals to arrive quicker at the sensors on the vertical tank walls. The simulated signal strength is affected by a combination of the accurately modeled outer layer windings and the reduced gaps between the adjacent disks of the MV winding. The observed differences in signal propagation characteristics significantly affect scenarios with short traveling times between in-winding sources and a sensor, meaning short distances. The effects decrease with an increase in elapsed time from the trigger of the PD source (increasing distances between the source and sensor).

### 3.2. Influence of Sensor Design

**Figure 12:**
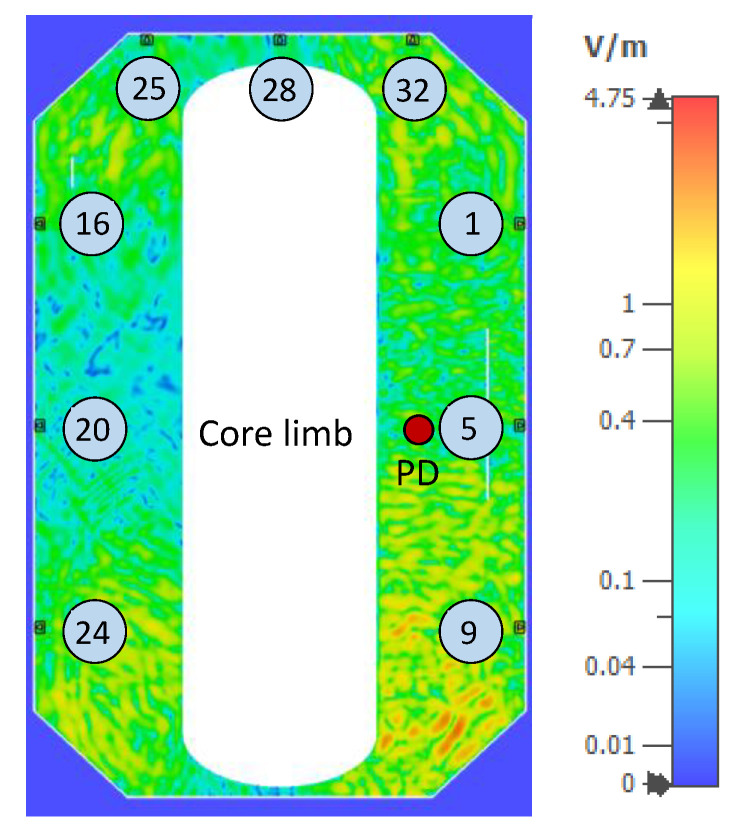
Electric field distribution on the first cutting plane at time *t* = 24 ns after PD source (red circle) trigger.

The time-domain simulations to study the EM wave propagation were performed in the frequency range of 0–900 MHz. In addition, the window-type sensor design with the short conductor was used in the simulations to keep the dimensions of the dielectric window consistent with the actual values. The transformer was divided into two-dimensional cross-sections by four cutting planes along the width of the tank, as shown in Figure 6, to analyze the electric field distribution. Each cutting plane passes through the mid-points of the sensors on that plane. As a result, all sensors can be conveniently analyzed. Additionally, to ensure that the electric field distribution was analyzed at a time instant where the EM waves had reached all sensors on a plane, the time-domain UHF signals were analyzed to pick a common time instant. First, the electric field distribution in the drain valve sensor design is analyzed, and thereafter, the similarities and differences in the field distribution between the window-type design are discussed. In the following analysis, the sensors located on the first cutting plane are evaluated, and the PD source is located in position U4, as shown in Figure 12. The analysis has been performed for all 12 PD sources and all four cutting planes, and the results were found to be similar in each case.

**Figure 13:**
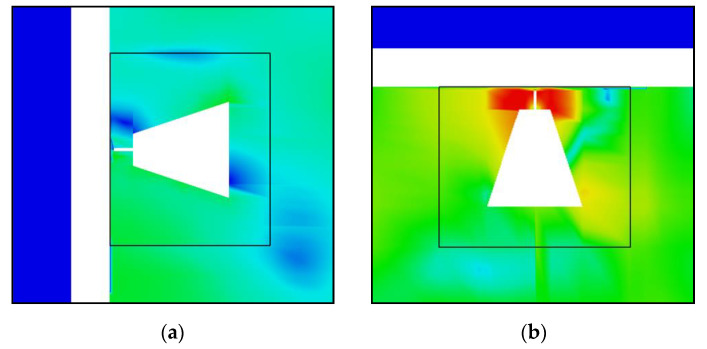
Electric field distribution around drain valve sensors. (**a**) Sensor 20; (**b**) sensor 32.

**Figure 14:**
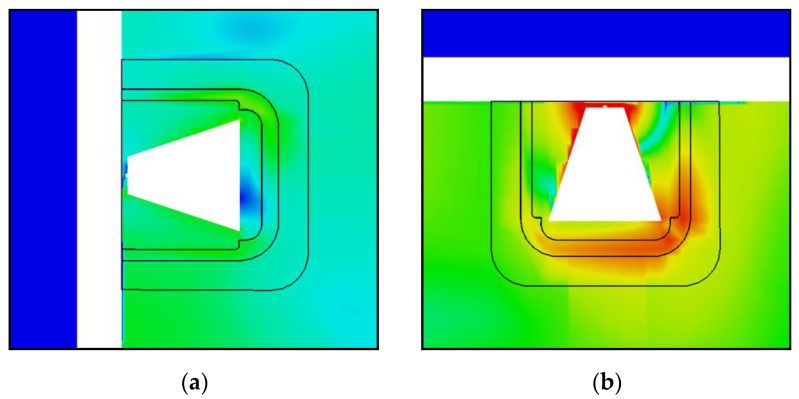
Electric field distribution around window-type sensors. (**a**) Sensor 20; (**b**) sensor 32.

The first cutting plane consists of sensors 1, 5, and 9 on the front tank wall; sensors 16, 20, and 24 on the rear tank wall; and sensors 25, 28, and 32 on the top of the tank, as shown in Figure 12. At time *t* = 24 ns from the trigger of the PD source, it was determined that the EM waves had reached all sensors on the cutting plane, and the electric field distribution on the cutting plane at this time instant is shown in Figure 12. The color ramp showing the electric field strength is the same for Figure 12, Figure 13 and Figure 14.

At *t* = 24 ns, the analysis of the electric field strength in the plastic cover of the drain valve sensor design showed that the lowest field strength was observed in sensor 20, with a value of 0.3 V/m near the front of the sensor head, as shown in Figure 13a.

The highest field strength was observed in sensor 32, where the area behind the base of the sensor head showed significant field enhancement at approximately 6 V/m, as shown in Figure 13b. This phenomenon of field enhancement behind the base of the sensor hear is typical of the sensors installed on the top of the tank. The degree to which the field is enhanced varies between the sensors and also depends on the PD source location and the time of observation. However, this phenomenon is not visible in the sensors installed on the front and rear tank walls.

An explanation of this phenomenon can be provided by observing the propagation of the EM waves, which have a direct path to the top of the tank (hence, higher electric field strength). The superposition of the EM waves in the narrow region between the base of the sensor head and the top of the tank leads to field enhancement. Additionally, the maximum electric field strength near the front of the sensor head was recorded to be 1.4 V/m.

The same sensors, as in the case of the drain valve sensors (sensors 20 and 32 respectively), exhibit the lowest and highest electric field strengths in case of the window-type sensors. In sensor 20, the maximum electric field strength is approximately 0.5 V/m in the air gap between the plastic cover and the dielectric window, as shown in Figure 14a, and it is 67% higher than that of its drain valve counterpart.

In the case of sensor 32, the maximum field strength, as shown in Figure 14b, is again in the area behind the base of the sensor with a value of 12 V/m, which is twice the value observed in the drain valve counterpart. The width of the gap between the base of the sensor head and the top of the tank is smaller in the window-type sensors, demonstrating that the width is inversely proportional to the electric field strength. Additionally, electric field enhancement is observed in the air gap, where a maximum field strength of approximately 4 V/m is recorded, which is 185% higher than the value recorded near the front of the sensor head of the drain valve counterpart. It was observed that the field enhancement is not uniform across all sensors and can change based on the sensor location, PD source location, and time of observation. However, it is evident that the window-type sensors experience higher fields in the air gap compared to those in the plastic cover of the drain valve counterparts.

On comparing the window-type sensors, the sensors installed on the top of the tank can experience up to 8 times higher field strengths in the air gap than their counterparts on the vertical tank walls. The explanation for higher field strengths in the sensors installed on the top of the tank remains unchanged.

**Figure 15:**
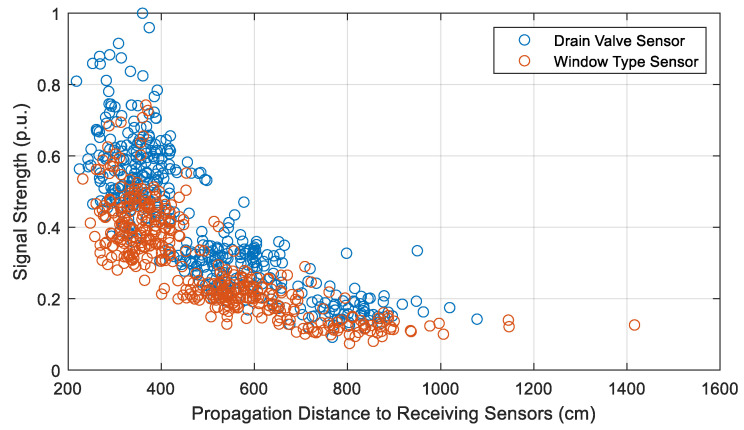
Comparison of the attenuation characteristics of the two sensor designs across different propagation distances.

Comparing the characteristics of the signals received (from the 12 sources inside the winding) by the two sensor designs, it is found that ToAs are quicker by approximately 0.61 ns in the case of drain valve sensors. This result is expected as the sensors are inserted further into the oil volume of the tank than the window-type sensors. As expected, the distance-dependent attenuation of the signals remains almost unchanged across the two designs, as shown in Figure 15, since the sensor and source positions remain unchanged, i.e., the propagation path remains unchanged. The signal strengths are not compared, since the window-type sensors have a lower insertion depth into the tank because of the dimensions of the dielectric window.

The primary difference between the two sensor designs is the field enhancement experienced by the window-type sensors in the air gap of the dielectric window when receiving UHF signals, which is expected because of the reduction of relative permittivity in the air gap. Any such field enhancement caused by the 50/60 Hz supply can be reduced by changing the insertion depth of the drain valve sensors (albeit at the cost of sensitivity), which changes the distance from the active parts. However, this is not possible in the case of the window-type sensors. Therefore, proper sensor positioning is of higher importance in case of the window-type sensors.

## 4. Conclusions

In this contribution, the influence of transformer winding design and UHF sensor design on the characteristics of the simulated signals has been studied. Three different types of windings were tested in a simplified single-phase simulation model, and the time of arrivals and propagation paths were analyzed. It was observed that broadband electromagnetic waves of UHF PD signals could propagate through very narrow gaps, which led to a difference in ToAs across the different winding models. Thereafter, the different winding designs are simulated in a model of an entire 300 MVA 420 kV transformer. It was found that the ToAs are generally quicker when the outer layer windings are properly modeled. Additionally, the strength of the signals received by sensors on the top of the tank increases when the layer windings are accurately modeled, and the MV winding does not have a gap between two adjacent disks. On properly modeling the outer layer windings, it was also observed that the difference in the signal propagation characteristics between the reference winding design were most pronounced just after the start of the PD event but slowly became less pronounced as the signals propagated through the tank. The differences in propagation characteristics were significant enough to consider accurate modeling of the windings even though the computational cost is increased. The distance-dependent attenuation of the UHF signals is also analyzed by considering both the propagation distance and the simplified LoS distance. It was found that when the PD sources were inside the winding, there were significant differences between the two methods. However, in the case of PD sources outside the winding, the propagation distance was approximately equal to the LoS distance, thus resulting in similar attenuation regardless of the method.

In addition, two different sensor designs, a drain valve design and a window-type design, were modeled and analyzed based on numerical simulations, which focused on evaluating the attenuation of the signal received by the sensors and the electric field distribution around the sensors when under operation. When the propagation path of the UHF PD signals remains unchanged, the two sensor designs exhibit the same distance-dependent attenuation characteristics. Studying the electric field distribution in the sensor designs showed that the window-type sensors experience higher electric field strengths than their drain valve counterparts because of the change in permittivity of the materials involved, specifically in the air gap between the sensor and the dielectric window. Therefore, the proper positioning of sensors in areas of low electric field stress, while necessary for both sensor designs, becomes more important in the case of the window-type sensors.

## 5. Outlook

In the next step, an experimental setup will be built to analyze the propagation of the EM waves through narrow gaps for simulation validation. Additionally, improved designs for the window-type sensors will be investigated.
